# The relation between neuromechanical parameters and Ashworth score in stroke patients

**DOI:** 10.1186/1743-0003-7-35

**Published:** 2010-07-27

**Authors:** Erwin de Vlugt, Jurriaan H de Groot, Kim E Schenkeveld, J Hans Arendzen, Frans CT van der Helm, Carel GM Meskers

**Affiliations:** 1 Department of Biomechanical Engineering, Faculty of Mechanical Engineering, Delft University of Technology, Mekelweg 2, 2628 CD, Delft, The Netherlands; 2 Department of Rehabilitation Medicine, Leiden University Medical Center, Albinusdreef 2, 2333 AL, Leiden, The Netherlands; 3 Laboratory for Kinematics and Neuromechanics, Departments of Rehabilitation Medicine and Orthopaedics, Leiden University Medical Center, Albinusdreef 2, 2333 AL, Leiden, The Netherlands

## Abstract

**Background:**

Quantifying increased joint resistance into its contributing factors i.e. stiffness and viscosity ("hypertonia") and stretch reflexes ("hyperreflexia") is important in stroke rehabilitation. Existing clinical tests, such as the Ashworth Score, do not permit discrimination between underlying tissue and reflexive (neural) properties. We propose an instrumented identification paradigm for early and tailor made interventions.

**Methods:**

Ramp-and-Hold ankle dorsiflexion rotations of various durations were imposed using a manipulator. A one second rotation over the Range of Motion similar to the Ashworth condition was included. Tissue stiffness and viscosity and reflexive torque were estimated using a nonlinear model and compared to the Ashworth Score of nineteen stroke patients and seven controls.

**Results:**

Ankle viscosity moderately increased, stiffness was indifferent and reflexive torque decreased with movement duration. Compared to controls, patients with an Ashworth Score of 1 and 2+ were significantly stiffer and had higher viscosity and patients with an Ashworth Score of 2+ showed higher reflexive torque. For the one second movement, stiffness correlated to Ashworth Score (r^2 ^= 0.51, F = 32.7, p < 0.001) with minor uncorrelated reflexive torque. Reflexive torque correlated to Ashworth Score at shorter movement durations (r^2 ^= 0.25, F = 11, p = 0.002).

**Conclusion:**

Stroke patients were distinguished from controls by tissue stiffness and viscosity and to a lesser extent by reflexive torque from the soleus muscle. These parameters were also sensitive to discriminate patients, clinically graded by the Ashworth Score. Movement duration affected viscosity and reflexive torque which are clinically relevant parameters. Full evaluation of pathological joint resistance therefore requires instrumented tests at various movement conditions.

## Background

Increased mechanical resistance to an imposed movement is common after central nervous system damage, such as stroke and may interfere with function. Its assessment and treatment are therefore major goals in rehabilitation. Main contributors to increased joint resistance are increased viscosity and stiffness of muscle and connective tissue (clinically labeled "hypertonia") and hyperactivity of the stretch reflex (clinically labeled "spasticity") [1]. The Ashworth Score (AS) is a widely used clinical measure of joint resistance [2]. The AS subjectively grades the manual sensation of mechanical resistance experienced by the examiner during a one second (1 s) joint rotation over the full range of motion [3]. The impossibility to discriminate between the underlying mechanisms and the limited reproducibility and resolution have been the motivating challenge to develop an alternative method describing joint resistance in quantitative neuromechanical measures from the torque response [4]. Discerning muscular and connective tissue properties from the neural reflexes would facilitate the diagnosis of the physiological substrate of increased joint resistance and the subsequent indication for treatment.

Quantitative studies focused on the characteristics of the torque response signals, either versus time or joint angle [2,5-7]. Peak torque, rate of change and offset of the torque were found to correlate with AS but did not allow for discrimination between individual components of joint resistance. Alternatively, computational models allowed for simultaneous estimation of viscosity, stiffness and reflex torque [8-11]. Critical in such model-based system identification is the structure of the model comprising the relevant neuromechanical components. As in almost any biological system, joint mechanical behavior is highly nonlinear for substantial changes of states, i.e. joint position and velocity, as is the case during e.g. an Ashworth test [12-14]. This implies that a specific linear model structure that is valid for one combination of states will be invalid for almost any other combination. As a consequence, results obtained from small amplitude models [8,14] may not be generalized to large amplitude conditions. For large amplitude joint rotations, important nonlinear properties such as e.g. the joint angle-dependent stiffness may not be neglected [9]. It is therefore not surprising that different and sometimes conflicting results were reported from different models and types of joint movements [2,8,9]. For a valid description of joint neuromechanical behavior during large angular excursions, nonlinear modeling is thus required.

The main goal of this study was to quantify the independent neuromechanical determinants of ankle joint resistance, i.e. muscle and connective tissue related stiffness and viscosity and reflex generated torque of stroke patients and healthy controls for a range of different movement durations using a nonlinear neuromechanical model. We then aimed to answer the following questions:

1. To what extent does duration of an imposed movement affect neuromechanical parameters, i.e. stiffness, viscosity and reflexive torque, in chronic stroke patients and healthy subjects?

2. Do neuromechanical parameters discriminate between stroke patients and healthy subjects?

3. Do neuromechanical parameters correlate to disorder severity as graded by the AS?

The clinical relevance of the instrumented identification is to directly attain patients to the appropriate treatment and to be able to quantify the effects of treatment.

## Methods

### Subjects & patients

A convenience sample of nineteen stroke patients (mean age 63.6, SD 8.5 years) was recruited from the outpatient clinics of the Department of Rehabilitation Medicine of the Leiden University Medical Center and the Rijnland's Rehabilitation Center, Leiden, the Netherlands. Patient demographics are summarized in Table 1. Inclusion criteria were unilateral stroke resulting in a hemi-paresis and the ability to walk a minimum distance of 6 meters. The use of an assistive device (cane or AFO, see Table 1) was permitted. Patients were excluded if they had severe cognitive or language deficits interfering with the comprehension of instructions required to participate in the study (Minimal Mental State Examination, MMSE < 25 points), a pre-existing walking disability and/or orthopedic problems of the paretic foot/ankle. Pre-existing walking disability was defined as a denial to the question "could you walk normally before the stroke?".

**Table 1:** Patient demographics

ID	Age	Sex	Lesion	Post stroke Time (months)	Ashworth Score	Spasmolytic medication	AFO/Cane
1	54	M	Hemorrhage R	16	3	-	-
2	78	M	Ischemia L	9	1	Diclofenac	-
3	61	M	Ischemia L	7	0	-	-
4	66	M	Ischemia R	15	0	-	-
5	82	M	Ischemia R	9	1	-	AFO
6	65	M	Ischemia R	16	0	-	-
7	53	M	Hemorrhage L	13	3	-	AFO
8	57	M	Ischemia R	15	0	-	-
9	59	M	Ischemia L	12	2	-	AFO/Cane
10	63	M	Ischemia L	8	1	-	-
11	54	M	Hemorrhage R	10	0	-	-
12	71	M	Ischemia L	6	1	-	-
13	70	M	Hemorrhage R	11	1	-	Cane
14	64	M	Ischemia R	11	0	-	Cane
15	56	M	Ischemia R	8	1	-	-
16	65	M	Hemorrhage L	7	3	-	-
17	51	M	Ischemia L	12	0	-	AFO/Cane
18	70	F	Ischemia R	12	0	-	-
19	69	M	Ischemia L	13	1	-	-

Seven healthy subjects (mean age 55.4, SD 10.3 years) were recruited as a control group. The medical ethics committee of Leiden University Medical Center approved the study. All participants gave their written informed consent prior to the experimental procedure.

### Instrumentation

**Figure 1:**
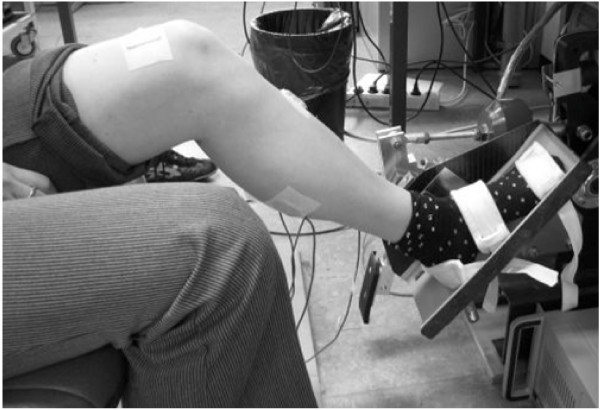
**Measurement set-up**. The subject's ankle was fixated on the footplate that was rotated by an electrically powered single axis actuator. Ankle reaction torque, ankle angle and EMG were measured during imposed ramp-and-hold movements.

Subjects were seated with their hip and knee positioned at approximately 110° and 160° of flexion respectively. Ankle rotations were applied by means of an electrically powered single axis footplate (MOOG FCS Inc., Nieuw Vennep, The Netherlands), see Figure 1. The foot was fixed onto the footplate by Velcro straps. Axes of the ankle and footplate were aligned by visually minimizing knee translation in the sagittal plane while rotating the footplate. Foot reaction torque was measured by means of a force transducer (Interface 1210AE-5000, resolution < 0.1 N, positive for plantar flexion torque). Angular displacement of the footplate was measured by a potentiometer at the footplate axis (Veccer S1998-1000 LB, resolution < 0.01 deg., positive for dorsiflexion direction). The motor was operated to impose either torques to assess ankle Range of Motion (RoM) or position for the ramp-and-hold (RaH) measurements to the subject.

Muscle activation of the tibialis anterior (TA), gastrocnemius lateralis (GL), soleus (SL) and gastrocnemius medialis (GM) was measured by electromyography (EMG) using a Delsys Bagnoli 4 system. Inter electrode distance was 10 mm. EMG signals were sampled at 2500 Hz, on-line band pass filtered (20-450 Hz) and off-line rectified and integrated by low pass filtering (3^th^-order Butterworth) at 20 Hz (IEMG). Reaction torque and ankle angle were sampled at 250 Hz. Angular velocity and acceleration were derived by single and double differentiation of the recorded angle signal respectively. To avoid amplification of noise due to differentiation, angle and force signals were low pass filtered at 20 Hz (3^th^-order Butterworth).

### Protocol

#### 1. Clinical test

Measurements were performed on the affected ankle of each patient and at the right ankle in case of controls. The Ashworth Score (AS) of the affected ankle [3] was assessed by an experienced physician [HA]. In order to avoid obtaining a biased and a study-specific Ashworth test, the physician was instructed to perform the Ashworth test as he would perform as usual in the clinic. Total time to perform the Ashworth test including positioning and instructing of the patient was about 5 minutes. The instrumented rotation measurements were performed by an experimenter [KS] who was blind to the clinical outcome. Judgment on the validity of the model was solely based on the recorded signals (internal validity). For the control group, only the instrumented measurements were performed. All measurements were completed within a single session of approximately one hour.

#### 2. Instrumented joint rotation

The ankle angle was defined as the position of the foot with respect to the lower leg; the perpendicular position was defined as zero degrees or central position. Maximum dorsiflexion angle was assessed by a monotonically in- and decreasing dorsiflexion torque (100 s up, 100 s down) imposed by the manipulator from zero to a maximum value of 15 Nm resulting in slow rotations of approximately 0.5 deg/s. The angle before onset of the dorsiflexion torque was taken as the maximal plantar flexion angle. The angular excursion in plantar flexion direction was limited to -30 degrees, which was the maximal angle of the manipulator. RoM was defined as the difference between the maximum dorsiflexion and plantar flexion angle and used as boundary for the subsequent RaH rotations. At 15 Nm the foot was approximately at a perpendicular angle with respect to the horizontal for all subjects. Consequently, the variability in torque introduced by gravity around the maximal dorsiflexion angles could be considered negligible and thus there was no need to compensate for gravity during these tests.

RaH rotations were performed by the manipulator through the full RoM at four different durations of 0.25, 0.5, 1 and 2 s. As RoM differed between subjects while durations were fixed, rotation velocities were different between subjects. Prior to each RaH rotation, the ankle was moved from central position to the maximal plantar flexion angle in 2 s time. Subsequently, at a random time instant but within 3 to 4 s, the RaH rotation was started. In all cases, the RaH rotation ended at the maximal dorsiflexion angle. The hold phase lasted for 4 s after which the ankle was moved back again to the central position. Time to cover a complete movement profile did not exceed 15 s. Rest periods of 30 s were maintained between each movement profile which is sufficient for full recovery of passive stiffness [15]. All movement profiles were performed twice to test for repeatability of the estimation procedure. Subjects were asked to remain maximally relaxed during the entire experiment and not actively resist any motions. Level of relaxation was checked off-line from EMG activity of all muscles prior to the RaH rotation. When IEMG was larger than three times standard deviation for longer than 1 s the observation was discarded from the analysis.

### Neuromechanical model, parameter estimation and internal validity

A neuromechanical computational model was used to simulate the total generated ankle torque. The model included a passive and an active muscle element, the latter being a Hill-type muscle model (see Appendix). The Achilles tendon was assumed to be infinitely stiff (see Discussion). The recorded ankle angle and IEMG signals were input for the model. The model was fitted to the total measured ankle torque defined within a time frame starting from 0.5 s before ramp onset until 0.5 s after the start of the hold phase. The model parameters where estimated for each single trial by minimizing the quadratic difference (error function) between the recorded and simulated ankle torque. Parameter estimation and analysis were performed in Matlab (The Mathworks Inc., Natick MA). In total ten model parameters were estimated which are summarized in Table 2.

**Table 2:** Model parameters

Parameter	Unit	Description	Initial Value	Estimated Value (mean ± 1 s.d.)
*m*	kg	mass (ankle + footplate)	2	1.86 ± 0.42
*b*	Ns/m	viscosity coefficient	5	1.28 ± 1.08
*k*	1/m	stiffness coefficient	100	26.4 ± 15.4
*x*_0_	m	muscle length shift	0	-0.0081 ± 0.0023
*F_0_*	N	muscle force shift	-25	-21.2 ± 9.6
*e_1_, e_2_*, *e_3_, e_4_*	N/Volts	EMG weighting factors	10000	3.5 ± 1.05, 2.0 ± 0.96, 3.1 ± 0.77, 2.6 ± 1.1 (× 10^5^)
*f*	Hz	activation cutoff frequency	1.5	1.28 ± 0.34

Model parameters, initial values used for estimation and estimated values (mean and standard deviation of all conditions and subjects).

The covariance matrix *P *was derived to determine the interdependence of the model parameters [16]:

where *N *is the number of time samples used for estimation of the parameters, *J *the Jacobian matrix, and *e *the 1 × *N *error vector. The Jacobian is a *N *× *n_p _*matrix, with *n_p _*= 10 the number of estimated parameters, containing first derivatives of the (final) error to each parameter.

Two different type of indicators were derived from the covariance matrix. The first is the *interdependence *of the parameters for which the auto-covariance (diagonal terms of *P*) of each parameter was compared to the cross-covariance (off-diagonal terms of *P*) between the one parameter and all the others. If the auto-covariance was higher than all cross-covariances, the corresponding parameter was estimated/assumed independently and its estimated value was assumed to be reliable. The second measure is the *sensitivity *of the parameters for which the auto-covariance value on itself is representative. High sensitivity means that the parameter has an observable contribution in the system's response (i.e. the ankle torque in this study) and therefore can be estimated with certain accuracy. The square root of the auto-covariance, such as obtained from *P *in the above expression, is the standard error of the mean (SEM) of the parameter estimation [16]. For high sensitivity, the SEM needs to be low compared to the corresponding parameter value.

For visual inspection, we have normalized the covariance matrix by dividing each *i,j*-th element by  (*i, j *from 1 to *n_p_*) such that all diagonal terms equal to one. SEM values were normalized to their corresponding parameter values and subsequently averaged over all trials and subjects.

Reproducibility of the parameter estimation was assessed by taking the difference of the two parameter values (one repetition) divided by their mean. Model internal validity was assessed by calculating the Variance Accounted For (VAF, "goodness of fit") describing the remaining difference after model optimization between simulated and measured ankle torque:

with *T_meas_*(*t*) the measured ankle reaction torque and *T_mod_*(*t*) the estimated ankle torque from the model (Eq. A1, Appendix) over the time frame used for parameterization.

As a measure of the amount of reflex activity, the root mean square (r.m.s.) of the modeled reflex torque was calculated over the time frame used for parameterization. The r.m.s. reflex torque from the triceps surae was derived from the corresponding reflex force (Eq. A15, Appendix) and moment arm (Eq. A5, Appendix) according to:

and similarly for the reflex torque of the tibialis anterior, with *n *indicating the time sample of the identification time frame [1 ... *N*]. The r.m.s. value is a common way to denote the energy of a signal.

The model parameters were defined on the (metric linear) muscle level while for interpretation and analysis of the results, viscosity and stiffness were expressed in the (angular) joint domain according to Eqs A10 and A11 (Appendix). Viscosity and stiffness increase exponentially with joint angle (muscle length). Because of the exponential relationship, both viscosity and stiffness could only be compared at the same joint angle, *θ*_comp_, for all subjects (controls and patients). *θ*_comp _was determined by the smallest maximal dorsiflexion angle amongst all subjects. Any differences in viscosity and/or stiffness between subjects and patients was largest at *θ*_comp_. Statistical testing of viscosity and stiffness at smaller joint angles was therefore considered less meaningful, hence not performed.

### Statistical analysis

For statistical analysis, a disease gradation was defined, ranging from healthy subjects to patients graded by AS. Thus, within the tested population, four groups were discerned, i.e. controls (C), a clinically unaffected patient group: AS0; a mildly affected patient group: AS1; and a severely affected patient group, i.e. the patients exhibiting an AS of 2 and higher: AS2+.

To test the differences in RoM between patients graded by AS and controls, a one way ANOVA was used with a Bonferroni post hoc test. Movement *duration *and *velocity *were separately related with the RoM. As RoM differed between subjects, *duration *and *velocity *were not interchangeable. Movement *duration *was standardized and thus the factor *duration *(not *velocity*) was applied in the analysis. To test the effects of movement *duration *and disease gradation, a Linear Mixed Model was used with disease gradation as fixed and movement *duration *as repeated factor. In case of significant effects of either factor, a Bonferroni post hoc test was used to specify the differences between the groups. Correlation between relevant neuromechanical parameters and AS was assessed using linear regression. All statistical testing was performed using SPSS 16.0, SPSS Inc. at an alpha of 0.05.

## Results

Both Controls and Patients could perform the tests. No problems were observed with cognitive or language deficits interfering with the comprehension of instructions required to participate in the study. A total of 10 trials from three healthy subjects were removed from the analysis because of sudden and large IEMG bursts of all muscles before the onset of the RaH movements, indicating insufficient relaxation.

### Range of Motion (RoM)

**Figure 2:**
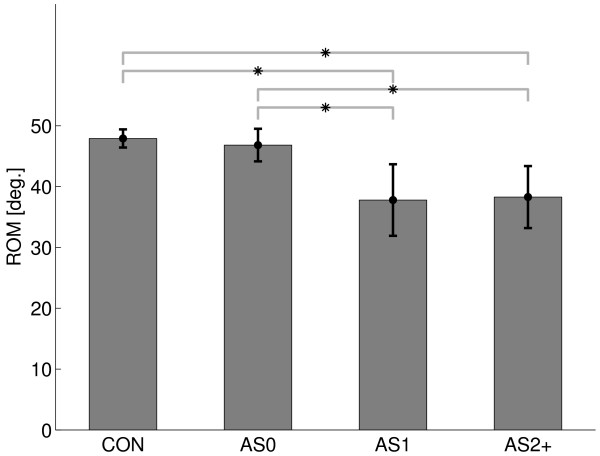
**Range of motion**. Range of motion (RoM) of all subject groups (mean and standard deviation). The asterisk denotes significant difference (see Results).

RoM differed between groups (F = 10.7, p < 0.001), see Figure 2. RoM was significantly smaller for the AS2+ group versus both the AS0 and control group and for the AS1 versus both AS0 and control group. The smallest maximum dorsiflexion angle amongst all subjects was *θ*_comp _= 3.03 degrees and was used for comparison of joint viscosity and stiffness between subjects.

All patients and controls reached to the maximal plantarflexion angle of -30 degrees, which was the limit of the manipulator. Consequently, all the observed loss in RoM was accounted for by the reduced dorsiflexion.

To check for stretch induced muscle activity that might have affected the RoM measurement, the mean IEMG at zero torque (before dorsiflexion torque was imposed) was compared to the mean IEMG at the maximal dorsiflexion torque. Mean IEMG was taken over a 1 s interval and was larger at 15 Nm than at zero torque for almost all subjects. However, the increments were small (0.5-1%) relative to the magnitude of the IEMG responses observed during the RaH movements (see further). Therefore, the small IEMG increment during the RoM measurements were considered to have a negligible effect on the reported RoM values.

### Torque response to ramp-and-hold movement

**Figure 3:**
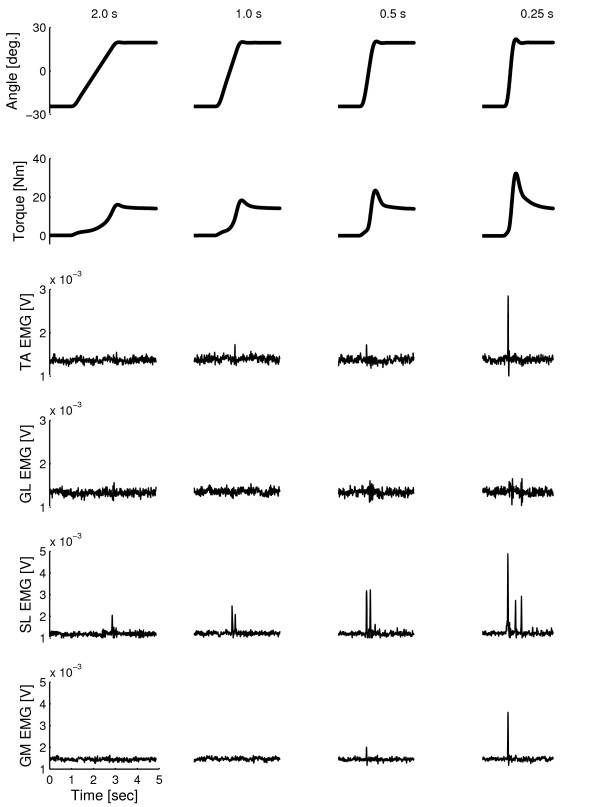
**Imposed ramp-and-hold movement profiles, joint torque and IEMG**. Rows from top to bottom: Ankle joint angle showing the imposed (dorsiflexion) ramp-and-hold (RaH) joint rotation profiles at four different movement durations (columns: 0.25, 0.5, 1.0, 2.0 s), corresponding joint torque responses and IEMG signals from all four muscles. Traces are shown over a five second time frame for an AS3 patient. Positive values indicate to dorsiflexion.

As an example, Figure 3 shows the imposed movement for all four durations and the corresponding torque and muscle activity (IEMG) of all muscles of a stroke patient (AS3). Torque typically increased exponentially during the ramp phase, reaching to a peak value near the end of the RaH movement. Peak torque increased with shorter duration (higher velocity) of movement. When the movement stopped at the dorsiflexion angle, the torque decayed to a value that was independent on duration.

Amongst all muscles, the soleus showed the highest activity in response to the imposed movements. Muscle activity emerged in brief bursts that increased in magnitude with shorter movement duration.

**Figure 4:**
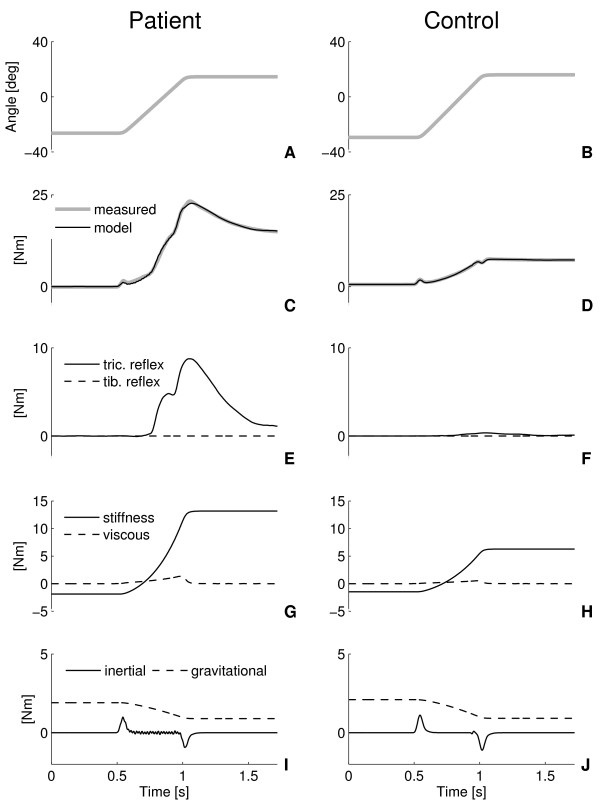
**Model fit**. Typical model fits at 0.5 s dorsiflexion duration. Left column: patient (AS3). Right column: control subject. A-B: imposed ankle movement; C-D: measured joint torque (grey) and torque as predicted from the model (black); E-F: reflex torque from triceps surae and tibialis anterior muscles; G-H; torque due to stiffness (solid) and viscosity (dashed); I-J: inertial (solid) and gravitational torque (dashed).

**Figure 5:**
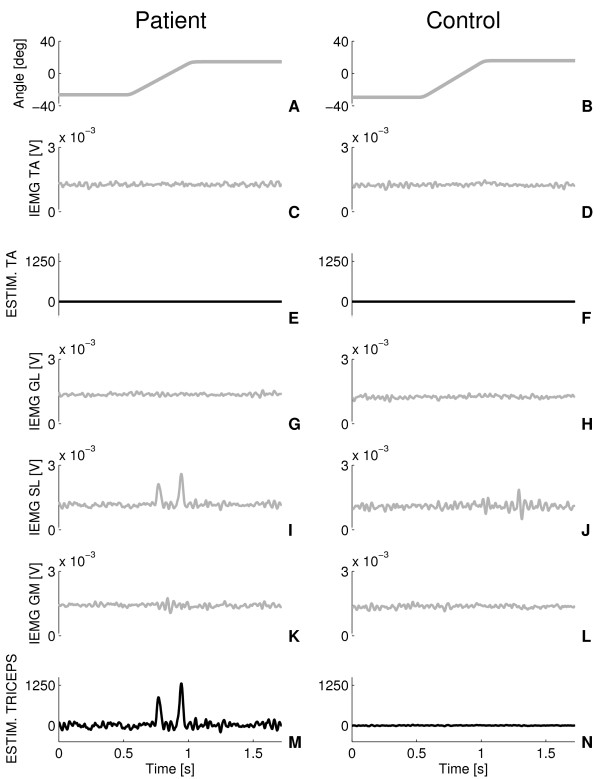
**Estimated IEMG activity**. Same patient (left column) and control subject (right column) and conditions as in Figure 4. Traces in grey are the IEMG signals from all muscles (C-D and G-L). The black traces (E-F and M-N) are the estimated (synthesized) muscle activity of the TA and triceps surae (sum of GL, SL and GM) respectively. The estimated signals were obtained from multiplication of the IEMG signals with the optimized weighting factors (*e*_1_-*e*_4_) and served as inputs to the muscle activation filters to produce the reflexive torque such as shown in Figure 4 (E-F).

Figure 4 shows a detailed view of the recordings (traces in grey) together with the model fits (traces in black). The measured torque (Figure 4: C, D) exhibited a brief inertial response at movement onset due to initial acceleration (Figure 4: I, J). Viscous, stiffness, inertia and gravitational torques are shown in Figure 4: G-J. Stiffness torque was observed at movement onset, increased rapidly during the ramp phase and sustained during the holding phase. Viscous torque was small compared to the stiffness torque (Figure 4: G, H). In both stroke patients and controls, IEMG activity of the triceps surae during the ramp phase was observed, generally consisting of one peak and occasionally followed by additional peaks (Figure 4: E and Figure 5: I). Reflex generated torque persisted for about 1 s due to the activation dynamics of the muscles (Figure 4: E, F). TA activity occurred in some cases at random time instances causing but a small dorsiflexion torque compared to the plantar flexion torque as generated by the triceps surae activity (Figure 4: E, F).

The composition of the net muscle activity from the individual IEMG signals is presented in Figure 5 (same subjects and conditions as in Figure 4; recordings in grey and model estimates in black). TA activity was absent. For the stroke patient, soleus activity showed distinct bursts and dominated the net estimated activity of the triceps surae. The estimated contribution of the three calf muscles to the total estimated reflexive torque (Figure 5 M), as obtain from the optimized weighting factors (*e_2, _e_3 _and e_4_*) was 3%, 91% and 6% for the GL, SL and GM respectively. Comparable distribution of muscle torque amongst the triceps surae was found for all other subjects and patients.

### Model validity and parameter accuracy

**Figure 6:**
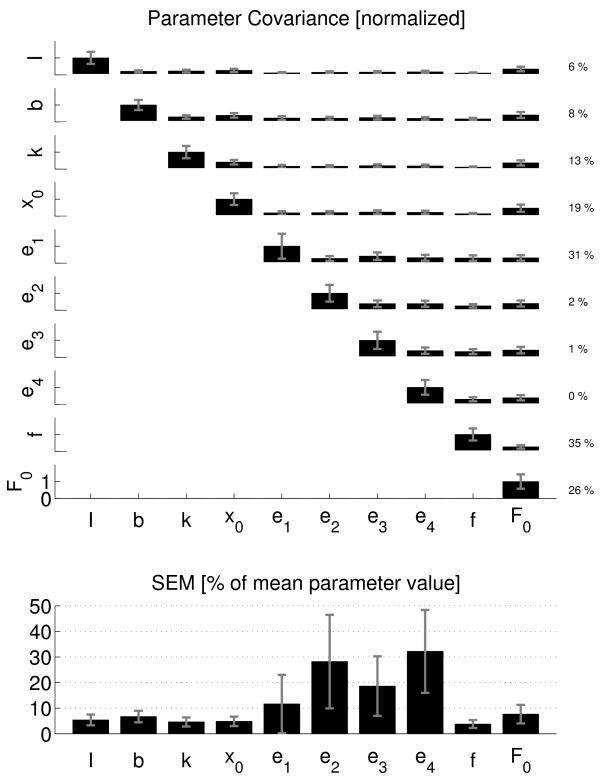
**Parameter covariance**. Covariance matrix *P *(top) and SEM values (bottom) of all estimated model parameters. Only the upper part of *P *is shown because of its symmetry. For normalization, see Method Section. Averages over all conditions and subjects (solid bars) ± 1 s.d. (grey error bars). The auto-covariance is on the diagonal of *P*. The off-diagonal terms of *P *are the relative cross-covariances between two different corresponding parameters. Percentages at the right are measures of interdependence, i.e. the number of times the auto-covariance was smaller than any of the corresponding cross-covariance values. The SEM is equal to the square root of the auto-covariance, divided by the corresponding mean parameter value.

The Variance Accounted For (VAF) was above 90% in all cases, meaning that the observed ankle torque could be well described by the model and the model structure was a valid representation of the dynamics of the ankle joint. The normalized parameter covariance matrix for all model parameters is visualized in Figure 6 (top). On the average, the auto-covariance (diagonal) was larger than the cross-covariance (off-diagonal) for all parameters, meaning that each parameter was estimated independently from the others, i.e. the interdependence was sufficiently low. The interdependence was expressed as the percentage (number of times) the auto-covariance was smaller than the corresponding cross-covariance values (Figure 6, next to each row at the right). For the mass, damping and stiffness parameters (upper four rows), the interdependence was smaller than 20%. The IEMG weighting factors showed even smaller interdependence (< 2%), with an exception for the TA weighting (31%). Interdependence of the activation cutoff frequency was highest (35%).

On the average, the SEM was less than 10% except for the IEMG weighting factors (Figure 6, bottom). The weighting factors of both gastrocnemii (*e_2 _*and *e_4_*) were least sensitive.

**Figure 7:**
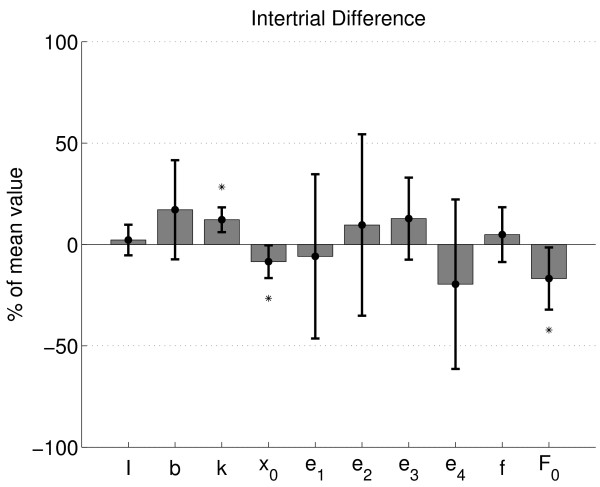
**Intertrial difference**. Intertrial parameter difference (solid bars: mean; error bars ± 1 s.d.) relative to the mean value of both measurements (one repetition), and then averaged over all conditions and subjects and for all parameters (horizontal axis). Asterisk denotes statistical difference from zero value.

Intertrial difference was less than 20% on average for all parameters, with exceptions for the IEMG weighting factors which showed larger differences (Figure 7). Viscosity and stiffness coefficients became smaller (positive difference) for the repeated measurements although only significant for the stiffness coefficient. Muscle length shift and force shift coefficients were larger (i.e. less negative values for the length shift parameter) with repetition. Intertrial difference for the mass and activation cutoff frequency were smallest (< 5%).

Estimated mass (1.86 ± 0.42 kg), muscle length shift (-0.0081 ± 0.0023 m), muscle force shift (-21.2 ± 9.6 N) and activation cut-off frequency (1.28 ± 0.34 Hz) did not change significantly with movement duration and also were not different between the patients and the control group. Viscosity and stiffness coefficients and reflex torque markedly differed as described in the following sections. Table 2 summarizes the initial and averaged (optimal) estimated values of all model parameters.

### Influence of movement duration

**Figure 8:**
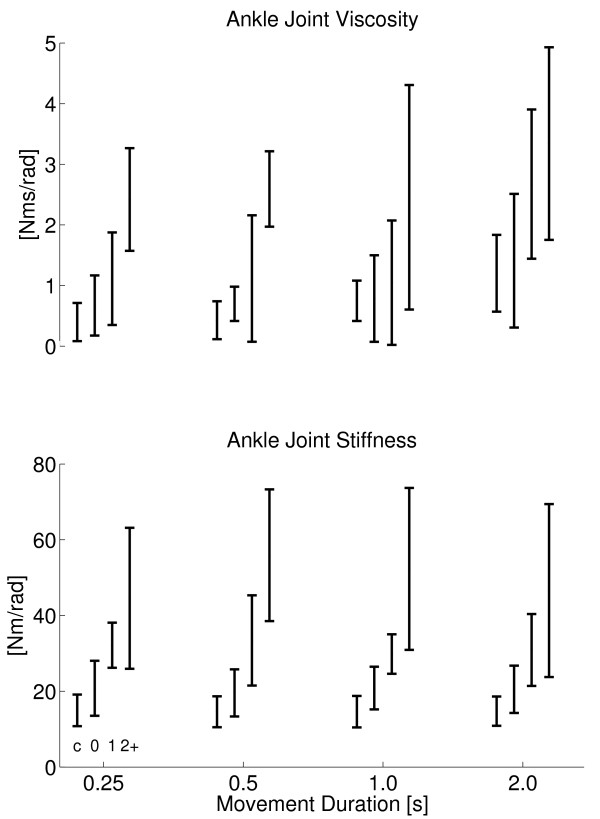
**Ankle Joint Viscosity and Stiffness**. Viscosity (top) and stiffness (bottom) for all subject groups against dorsiflexion duration. Subject groups (C, AS0, AS1, AS2+) from left to right for each cluster, denoted by c, 0, 1 and 2+ respectively. Joint viscosity and stiffness were taken at the same ankle angle for all subjects (controls and patients) being 3.03 degrees dorsiflexion (see Methods).

**Figure 9:**
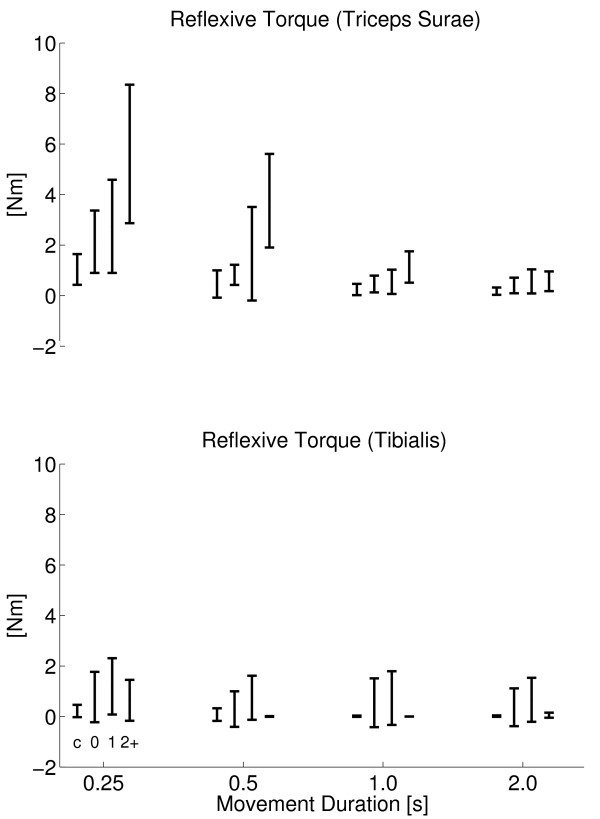
**Reflexive torque**. Stretch reflex torque (r.m.s.) for all subject groups against movement duration for triceps surae (top) and tibialis anterior (botttom) muscles. Subject groups (C, AS0, AS1, AS2+) from left to right for each cluster, denoted by c, 0, 1 and 2+ respectively.

Viscosity significantly increased with movement duration (F = 10.5, p < 0.0001). However, post hoc testing revealed that only for the 2 s duration viscosity was significantly larger (Figure 8, top). Reflexive torque (r.m.s) from the triceps surae (Figure 9, top) significantly decreased with movement duration (F = 56.3, p < 0.001). Stiffness was not affected by movement duration (Figure 8, bottom).

### Difference between patients and controls

Ankle viscosity (F = 20.2, p < 0.0001), stiffness (F = 19.5, p < 0.0001) and reflexive torque of the triceps surae (F = 5.8, p = 0.003) differed with disease grade. Post hoc testing revealed that for ankle viscosity and stiffness, control subjects could be discerned from stroke patients with an AS of 1 and higher; for reflexive torque, controls differed significantly from patients with an AS2+.

### Interaction of disease grade and test condition

Reflexive torque of the triceps surae decreased with duration and this effect was stronger for patients with higher AS (Figure 9, top, interaction term F = 2.91, p = 0.013). At the 1 s movement duration, stiffness significantly related to AS (r^2 ^= 0.51, F = 32.7, p < 0.001) while reflex torque did not (r^2 ^= 0.09, F = 3.22, p = 0.08). At shorter durations, reflex torque significantly related to disease grade (r^2 ^= 0.25, F = 11, p = 0.002). Reflex torque from tibialis anterior did not relate to movement duration nor to AS.

## Discussion

The overall aim of this study was to estimate neuromechanical parameters at the ankle joint in stroke patients during ramp-and-hold (RaH) rotations with different duration using a nonlinear dynamic ankle model. The experiments included the Ashworth test condition: a typical 1 s rotation over the full range of motion, which is clinically used to judge joint resistance in spasticity.

### Influence of movement duration on neuromuscular properties

Stretch reflex torque from the triceps surae showed a marked threshold in the movement duration in between 0.5 - 1.0 s, above which there was no substantial reflex response observed (Figure 9, top). The increase of reflexive torque from the triceps surae with movement duration beyond the threshold was expected for it is consistent with the well known velocity dependence of the stretch reflex [17].

The only other parameter that was influenced by movement duration, albeit slightly, was joint viscosity (Figure 8, top). The slower the joint was rotated the larger its viscosity (velocity to force relation). The increased viscosity was significant only for the longest (2 s) duration indicating to a nonlinear relationship.

### Difference between controls and patients

Stiffness, viscosity and reflexive torque from the triceps surae significantly differed between controls and the stroke patients with an AS of one and higher. Increased stiffness was not significantly higher for patients with AS0 compared to controls, indicating small differences with a statistical problem of power.

Although subjects were instructed to relax and not react to the RaH movements, stroke patients may have exhibited an increased ankle torque due to a possible higher background activity of the muscles at rest, as was reported by [18]. Also, an increase in stiffness from within the interior of the muscle cell was found in spastic muscle tissue and which is believed to originate from altered strain properties of intracellular proteins like titin [19,20]. We assumed that the increased stiffness in the stroke patients as found in this study was mainly from intracellular tissues since the observed stiffness behavior was well described by an exponential force-length relationship (Eq. A9) that is typical for passive tissues [13,21-23]. Increased stiffness at joint positions beyond the 'relaxed' position is believed to underlie contractures (muscle shortening) as observed in spastic patients [19,20].

### Disease severity is expressed by tissue stiffness in stroke

Intrinsic ankle stiffness was responsible for the increased AS in stroke patients. This means that joint resistance, as was indicated by the AS, is accounted for by the physical property 'stiffness', which is most likely originating from passive tissues. For the extent that AS provides a measure of disease severity, at least for the changes within the mechanical condition of the joint secondary to the neural disorder, we now may state that stiffness of the passive tissues increases with disease severity in stroke.

### Ashworth Scale does not comprises the stretch reflex response

Mechanical joint resistance is never determined by passive stiffness only, since reflexive torque was present during all applied RaH movements. However, for the two longest movement durations lasting 1 s, i.e. the Ashworth test duration, and 2 s the reflexive contributions were small. At shorter movement durations of 0.5 s and 0.25 s, the reflex torque from the triceps surae increased with AS.

### Ashworth test versus instrumented ramp-and-hold movements

It is important to realize that the manual performance of the Ashworth test may differ from the instrumented ramp-and-hold movements as applied in the present study. The instrumented conditions were of a constant velocity (ramp phase) whereas imposed manual manipulations may result in a bell-shaped velocity profile [24]. Therefore, the instrumented tests in this study are to be considered as separate tests next to the Ashworth test. Direct comparison to the Ashworth test must be taken with some care, but only for those properties that appeared to be dependent on movement velocity being joint viscosity and the stretch reflex torque, as was discussed above.

For the sake of direct comparison to the AS, movement duration was chosen to be the independent controlled variable, but resulted in different velocities between patients and controls. Thus, a structural bias with higher Controls velocities (because of increase RoM) was included in the inter-subject analysis of viscosity and triceps surae reflex torque. If velocity was controlled for, viscosity would likely exhibit less differences between controls and patients and less interaction with disease grade (AS). For the triceps surae reflex torque, the opposite would occur: differences between controls and patients, and in between AS groups, would be larger if velocity was controlled for. Although viscous torques have a marginal contribution to the overall joint torque in comparison to the stiffness and reflex torques, the bias problem requires the inter-subjective significance of (only) the tissue viscosity to be taken with care.

However, discrepancy between the description of the Ashworth test and the actual manual performance underlines the necessity of applying controlled test conditions to obtain reliable and valid outcome parameters.

### Validity of the method

The full model consisted of 10 parameters that were estimated reliably as indicated by the low interdependence values (Figure 6, top). For all but the IEMG weighting factors, the sensitivity was high (low SEM values). The combination of low interdependence and high sensitivity indicates that these parameters were estimated reliably and accurately.

Both viscosity and stiffness coefficients decreased 17% and 12% on average respectively with repetition. This decrease in passive joint visco-elasticity with ongoing loading was previously reported in both the normal and spastic case, e.g. [23,25,26]. Also, the length shift parameter was 9% larger with repetition which means that the ankle joint angle beyond which the visco-elastic torque started to increase shifted to dorsiflexion, which is probably related to the decrease in visco-elasticity.

The force shift coefficient (*F_0_*) had influence on all parameters (last column of the covariance matrix). Based on the small interdependence amongst most other parameters, it follows that the estimation of *F_0 _*was influenced by the other parameters to some extent. The prime role of *F_0 _*was to shift the exponential force-length characteristic to have more flexibility in describing the ankle stiffness but perhaps it was also used to account for small model remnants.

The IEMG weighting factors, in particular these for the gastrocnemii muscles, were least sensitive while their interdependence was exceptionally small. This means that the contributions of the gastrocnemii could be estimated independently but their estimated contributions to reflexive torque were far less compared to the soleus muscle. The intertrial difference for the soleus was smallest (12 ± 20%) which confirmed its dominant contribution to triceps surae reflex torque compared to the gastrocnemii muscles.

Because the gain (participation) of each EMG channel was also estimated, the method was free to select which muscles contributed and to what extent. Any cross-talk between agonists (soleus and both gastrocnemii) was therefore of no problem. Cross-talk between antagonistic muscles may have disturbed the selection between muscles. However, it has been shown that there is 5% cross-talk from the tibialis to the soleus at most and under supra maximal stimulation [27]. It was not likely that supra maximal activation occurred during our experiment so any effect of cross-talk was most likely very small.

In our model, the Achilles and tibialis tendons were taken as infinitely stiff. Over all subjects and patients plantarflexion torque never exceeded 30 Nm. In normal subjects maximal voluntary contraction (MVC) produces about 150-225 Nm (female-male) of plantarflexion torque at 10 degrees dorsiflexion [28]. Thus, plantarflexion torque was in the range of 13-20% MVC of normal, resulting in a maximal tendon elongation of 0.4-0.6 cm respectively [29]. The total muscle-tendon length change followed directly from the ankle angle and moment arm and varied in the range of 3.5-4.7 cm, which means that 17% of the muscle-tendon length change would be from the Achilles tendon at most. As the consequence of omission of the Achilles tendon in our model, joint stiffness and viscosity values may be slightly underestimated since we assumed one element instead of two elements in series. An infinitely stiff Achilles tendon has also been assumed in previous studies that estimated neuromechanical properties of the elbow and ankle joint [8,9,14,30].

Overall model validity is illustrated by high "goodness of fit" (VAF) values that were above 90% for all movement conditions tested. Together with the low interaction between the parameters and high sensitivity of the model parameters we therefore may conclude that the underlying neuromechanical behavior of the ankle joint was well quantified by the model for all conditions tested.

### Comparison to the literature

Increased stiffness was also observed in a comparable study [8] in the paretic limb of stroke patients, but which did not increase with AS. In that particular study, continuous (> 30 s) small amplitude joint rotations (1.5 degrees) were applied at high speeds. Since the joint system (as any biological system) is highly nonlinear [13,31] and varies as a function of time, long lasting small amplitude behavior cannot be generalized or extrapolated to brief (< 2 s) large amplitude behavior (> 15 degrees) as used in an Ashworth test and applied in the current study.

Ankle joint viscosity had a mean value of 0.69 Nms/rad and 1.14 Nms/rad for the control and patient group respectively, which are in the same ranges as found previously by [14]. Mean ankle joint stiffness was 14 Nm/rad for the control group and 31 Nm/rad for the stroke patients, which are both a factor 3 to 4 lower than found by [14] and for the controller group a factor 3 lower than found by [13]. The discrepancy can be explained from the usage of much smaller displacements (several degrees) in [13,14] as passive joint stiffness strongly increases with decreasing amplitude of displacement [13,31].

The mean estimated mass was 1.86 kg and modeled as a point mass at a fixed distance of 0.15 m from the rotation center of the ankle joint, i.e. the inertia was 0.042 Nms^2^/rad. The inertia of the footplate was 0.032 Nms^2^/rad such that the mean foot inertia was 0.010 Nms^2^/rad, which is only slightly higher than the range of 0.007-0.009 Nms^2^/rad as previously reported [32,33].

In previous studies, reflex contributions to ankle torque were estimated by quantification of parameters from a feedback model representing the functioning of the muscle spindles and Golgi tendon organs [8,14,34]. However, the inputs to proprioceptive sensors such as length (velocity) and force of the muscle fibers cannot be measured during movement experiments accurately to date. This study directly estimates reflex torque from measured muscle activity (IEMG) and therewith no assumptions about the functioning of the proprioceptive sensors were required, allowing for direct estimations of the net reflex torque.

Ankle stretch reflexes in stroke patients were previously found above angular velocities of 80 deg/s [29], which is comparable to the RaH rotations of 0.5 s duration (≈ 40 deg. ROM in 0.5 s) in this study. Rotation velocity during the Ashworth test, performed as a 1 s full RoM movement [3], is therefore assumed to be sub-threshold not evoking stretch reflexes.

The muscle length shift parameter *x_0 _*was used for shifting the exponential stiffness function with muscle length and can be interpreted as the muscle length at which the passive elastic force starts to increase substantially. The shift was -8.1·10^-3 ^m on average. In [13], the ankle angle at which passive plantarflexion torque started to increase rapidly was approximately 0.4 rad plantarflexion (-23 deg). For our model, the angle at which the passive stiffness torque started to increase was for that muscle length *x *where the exponential power term *x - x_0 _*(Eq. A8) was zero, that is for *x *= -*x_0 _*= 8.1·10^-3 ^m. From Eqs. A4 and A5 it follows that this value for *x *corresponded to an angle of -0.43 rad, which is close to the referred value above. The shift parameter can be interpreted as a physiological meaningful parameter describing the passive elasticity property of the triceps surae and was not different for the stroke patients compared to the controls. Apparently, the increase in passive tissue stiffness in the stroke patients was fully described by the (increased) curvature parameter *k *of the stiffness force-length relationship (Eq. A8).

Cut-off frequencies of second order models describing muscle activation dynamics have been reported in several previous studies. Most of these studies found values ranging from 1 to 3.3 Hz. In [14] maximal values around 7 Hz were found for the ankle triceps, which seems too high to our opinion. The mean value of 1.28 Hz as reported in our study is within the range of 1.0 - 1.4 Hz as found by [35] and somewhat lower than the cut-off frequencies found for the trunk (2.0 - 3.3 Hz) [36] and for elbow muscles (1.9 - 2.8 Hz) [37], likely because the soleus muscle is composed largely of slow twitch muscle fibers.

Muscle activity in response to the imposed (fastest) movements was observed as distinctive bursts (Figure 5: I) and were more pronounced for the stroke patients (not shown). Similar bursts of activity during comparable joint movements were reported by others [23,38,39]. Likely, the motoneurons in stroke patients tend to fire in a more synchronized way in response to afferent input from the stretch receptors that may be the result of decreased motoneuron thresholds [40] or increased sensitivity of afferent inputs [41].

In [42], a similar nonlinear relationship was found for the ankle in SCI patients with largest increase in viscosity below 20 deg./s, which is in the same range as the velocities during the 2 s movements (~ 40 deg.) in the current study. Viscous behavior of connective tissues (intra and extra muscular) [43] and a possible small amount of actin-myosin cross-bridges in the resting muscle [44] may have contributed to the velocity dependent behavior. The relationship between movement velocity and joint viscosity remains to be solved and may be important for understanding energy dissipation in functional tasks, e.g. during walking.

### Clinical implications

The current findings that joint viscosity and reflex torque depended on the duration, and thus the velocity of movement, implicate that for unambiguous assessment of joint resistance the Ashworth test should be performed in a strictly standardized way, actually according to a prescribed velocity instead of a 1 s movement. However, stretch velocity is difficult to standardize in manual testing. Instrumented evaluation comprising extended experimental conditions in combination with nonlinear computational modeling may prove to be a powerful tool to evaluate joint function.

Instrumented tests, like the one applied here, facilitate assessment of quantitative and objective ranges of neuromechanical properties correlating to disorder severity and may guide the clinician in optimal treatment planning e.g. choosing a stiffness reducing strategy instead of reducing reflex activity.

### Limitations

Functional evaluation, e.g. during walking, is compulsory for treatment guidance which can not be extrapolated from passive movements as studied here. We prepare for a larger study to compare neuromuscular properties as measured during static (sitting) and dynamic (walking) conditions to determine to what extent static measures can be used to predict functional improvement during dynamic conditions.

### Future research

Contribution to joint stiffness and viscosity from any muscle background activity could not be explicitly separated by the current model. That is, all angular velocity and angle related intrinsic torques where lumped together into a viscous and a stiffness torque component respectively. A further division between passive visco-elastic torque and torque emerging from (constant) muscle activation is planned for future studies. To determine its clinical value, we plan to apply the current method to a larger cohort of patients to study the effect of different interventions on neuromuscular properties of the ankle joint.

## Conclusion

This study demonstrated a new measurement technique for quantification of neuromechanical parameters of the individual ankle joint from a single dorsiflexion movement. Tissue and reflex torque were most sensitive parameters to discriminate stroke patients from healthy control subjects and also "grade" patients. Stroke patients exhibited increased ankle stiffness and viscosity with AS. For movement durations shorter than 1 s stroke patients also showed increased reflex torque with AS. Joint resistance observed during the 1 s movement over its RoM originated mainly from increased tissue stiffness. Correlations of relevant parameters to AS were assessed on group level and the relatively high standard deviations illustrate the difficulty experienced in discrimination between AS grades in the clinical practice.

The developed model fully covered the observed neuromechanical behavior of the ankle joint. It provides a basis for further dividing the visco-elasticity into contributions from connective and (active) muscle tissue, and the reflex torque into contributions from muscle spindles and Golgi tendon organs. The present study was primarily aimed at development of the method. Inclusion of larger and more divergent patient groups will demonstrate whether clinical phenotypes can be identified in (combinations of) abnormal system properties, such as enhanced stiffness and reflex torque. This may then be the foundation for therapy guidance, e.g. splinting, casting or surgery versus botulinum toxin. Establishing the sensitivity to interventions is a first step towards therapy evaluation.

We conclude that the combination of instrumented evaluation including multiple experimental conditions and nonlinear computational modeling is a powerful tool to quantitatively assess joint resistance. Objective and high resolution identification of neuromuscular parameters will be of use in daily clinical practice.

## Competing interests

The authors declare that they have no competing interests.

## Authors' contributions

EV designed the experiment, wrote the processing software, developed the mathematical models and wrote the manuscript. JG co-designed the experiment, assisted in the data processing and interpretation and writing of the manuscript. KS conducted the experiments and recruited the patients. HA took part in discussions on the outcome and critically reviewed the manuscript. FH took part in discussions on the outcome. CGM co-designed the experiment, assisted in the processing and interpretation of data and critically reviewed the manuscript. All authors read and approved the final manuscript.

## Appendix 1: Neuromuscular model

Ankle joint resistance is described by:(A1)

where *t *is the independent time variable [s], *T_mod _*the modeled ankle reaction torque [Nm],  the ankle angular acceleration [rad/s^2^], *I *the inertia of ankle plus footplate [kg.m^2^], *T_tri _*the torque generated by the plantar flexion muscles (GL, SL, GM), or triceps surae [Nm], *T_tib _*the torque generated by the dorsiflexion muscle (TA) [Nm], and *T_grav _*the torque due to gravity [Nm].

Although the TA was not substantially stretched during the ramp phases in the current experiment there was considerable reflex activity during some RaH movements. For these reasons, viscosity and stiffness were modeled for the plantar flexor muscles only and reflexive force was included for both plantar and dorsiflexion muscles.

Muscle torques were described by:(A2)(A3)

where *x *is the (change) of muscle length (linear displacement) [m],  the rate of change of muscle length [m/s], *F_visc _*the velocity related muscle force from tissue viscosity [Ns/m], *F_stiff _*the length related muscle force from tissue stiffness [N/m], *F_reflex,tri _*and *F_reflex, tib _*the reflexive muscle forces from the triceps surae and TA respectively [N], and *r_achil_*(*θ*) and *r_tib_*(*θ*) the angle dependent moment arm [m] of the Achilles tendon and tibialis anterior tendon respectively.

Triceps surae muscle length change was obtained from its moment arm:(A4)

Positive values for *θ *[rad] denote dorsiflexion direction, and thus positive values for *x *denote lengthening of the triceps surae. Achilles tendon moment arm (*r_achil_*) was assumed to scale linearly with joint angle, as derived from [45], according to:(A5)

The moment arm of the tibialis anterior tendon was described by [46]:(A6)

Inertia of ankle plus footplate was modeled as a point mass *m *[kg] at distance *l_a _*(fixed at 0.15 m) from the center of rotation, i.e.  [kg.m^2^]. Torque due to gravity equals:(A7)

where *θ*_*fgnd *_represents the angle of the foot with respect to the horizontal (ground) at zero degrees ankle angle [rad]. Here, *θ*_*fgnd *_equals to the angle the point mass had (around the ankle rotation axis) with the horizontal in central position and which was fixed at 10 degrees, and *g *is the gravitational acceleration (*g *= 9.8 m/s^2^).

The viscous and stiffness components were modeled as follows:(A8)(A9)

Force due to stiffness (Eq. A9) exponentially increases with ankle angle corresponding to the length tension properties of ligamentous, tendinous and muscular elastic tissues [12,23,47-49]. Increased tissue stiffness, as often seen in spasticity [20], can be described by Eq. A9 as a steeper (or shifted) force-length relationship. We assume viscous forces of tissues along the ankle joint to relate to compression (shear forces), which increase with tension. Therefore, both viscous and stiffness force scale with position (Eq. A8, A9). Exponential increase in viscous force with joint angle was also derived from [23]. The exponential curvature is shaped by *k *[1/m], called the stiffness coefficient, while the amount of viscosity is obtained by multiplication the same curvature with the viscosity coefficient *b *[Ns/m]. Two shift parameters are included in Eqs A8 and A9 such that the viscous and stiffness forces can be shifted in two dimensions, that is, in length by *x_0 _*and in force by *F_0_*. The muscle length beyond which the force starts to increase exponentially is determined by the shift parameter *x_0_*, a known property of passive muscle stiffness. The offset force term, *F_0_*, served purely as a shaping parameter for the stiffness model.

For comparison, joint viscosity, *B*_joint_, and joint stiffness, *K*_joint_, were taken at an angle that was the same for all subjects (*θ*_comp_) and equal to the smallest dorsiflexion angle amongst all subjects (see also Methods):(A10)(A11)

where *x*_comp _the triceps surae muscle length corresponding to *θ*_comp_.

Neural muscle activity for both tibialis and triceps surae due to stretch reflexes was estimated from corresponding IEMG signals according to:(A12)(A13)

where *u_tib _*and *u_tri _*the neural activity for the tibialis and triceps surae respectivly, *e_1 _*- *e_4 _*are weighting factors [N/Volts], numerical subscripts (1 - 4) correspond to the IEMG signals of the four muscles as referred to by subscripts TA, GL, SL and GM respectively.

The neural activity is then passed through a linear second order filter (equal for both muscle groups) describing the muscle activation process to produce the active state of the muscle [35,50]:(A14)

and similarly for the tibialis anterior, where α_*tri *_the active state of the triceps surae, *ω*_0 _= *2π f_0 _*the cutoff frequency of the activation filter, and *s *the Laplace operator denoting the first time derivative. The relative damping *β *was set to one (critically damped) [35].

A Hill-type muscle model was used to compute the muscle force from the active state and the muscle length and velocity according to:(A15)

and similarly for the tibialis anterior. For a full description of the structure and the parameters of the force-velocity and force-length relationships of the model (Eq. A15) we refer to [47]. The most important parameter values were: optimum muscle length triceps surae (tibialis) 3.5 (4.6) cm occurring at central ankle angle; maximum shortening velocity 8 (8) times optimum muscle length; maximum eccentric force was 1.5 (1.5) times the isometric force, and the isometric force was normalized to 1 since scaling of force was fully determined by the weighting factors *e_1_*-*e_4_*.
